# A 5-Minute Cognitive Assessment for Safe Remote Use in Patients With COVID-19: Clinical Case Series

**DOI:** 10.2196/26417

**Published:** 2021-06-14

**Authors:** Thomas Beresford, Patrick J Ronan, Daniel Hipp

**Affiliations:** 1 Laboratory for Clinical and Translational Research in Psychiatry Rocky Mountain Regional VA Medical Center Aurora, CO United States; 2 Department of Psychiatry School of Medicine University of Colorado Aurora, CO United States; 3 Research and Development Service Sioux Falls Veterans Affairs Medical Center Sioux Falls, SD United States; 4 Department of Psychiatry and Basic Biomedical Sciences Sanford School of Medicine University of South Dakota Sioux Falls, SD United States

**Keywords:** cognition, COVID-19, safety, remote use, delirium, brain injury, brain, diagnosis, assessment, test, telehealth, telemedicine

## Abstract

**Background:**

Early clinical experience during the COVID-19 pandemic has begun to elucidate that the disease can cause brain function changes that may result in compromised cognition both acutely and during variable recovery periods. Reports on cognitive assessment of patients with COVID-19 are often limited to orientation alone. Further assessment may seem to create an inappropriate burden for patients with acute COVID-19, which is characterized by fatigue and confusion, and may also compromise examiner safety.

**Objective:**

The aims of this study were to assess cognition in patients with COVID-19 as comprehensively as possible in a brief format, while observing safety precautions, and to establish a clear face value of the external validity of the assessment.

**Methods:**

We adapted a brief cognitive assessment, previously applied to liver transplant candidates and medical/surgical inpatients, for remote use in patients hospitalized for COVID-19 treatment. Collecting quality assurance data from telephone-administered assessments, this report presents a series of 6 COVID-19 case vignettes to illustrate the use of this 5-minute assessment in the diagnosis and treatment of brain effects. Primary medical teams referred the cases for neuropsychiatric consultation.

**Results:**

The age of the patients varied over four decades, and none of them were able to engage meaningfully with their surroundings on admission. On follow-up examination 6 to 10 days later, 4 of the 6 patients had recovered working memory, and only 1 had recovered calculation ability. Of the 6 patients, 2 were capable of complex judgment responses, while none of the cases completed frontal executive function testing in the normal range.

**Conclusions:**

Cognitive assessment in patients with COVID-19 using this remote examination reveals patterns of cognitive recovery that vary among cases and are far more complex than loss of orientation. In this series, testing of specific temporal, parietal, and frontal lobe functions suggests that calculation ability, judgment, and especially frontal executive functions may characterize the effects of COVID-19 on the brain. Used widely and serially, this examination method can potentially inform our understanding of the effects of COVID-19 on the brain and of healing from the virus.

## Introduction

Early clinical experience with the COVID-19 pandemic has begun to elucidate that the disease can cause brain function changes, resulting in compromised cognition. These changes occur both acutely and during variable recovery periods. High frequencies of delirium and other brain phenomena in patients with COVID-19 [1,2] require adequate assessment of the patients’ cognitive functions while preserving the health of caretakers. Safety is paramount for all caregivers, especially those with risk factors such as advanced age or physical vulnerability to the respiratory effects of COVID-19. Early literature reports, however, present only minimal cognitive assessments that are frequently confined to orientation [3]. A more complex assessment of specific cognitive functions enables better characterization of the extent to which factors such as the “cytokine storm” or other forms of inflammation, concurrent delirium, stroke, or virus infection of the brain may be at work in the course of recovery [4].

We defined three characteristics for a safe, workable cognitive assessment: (1) brevity, (2) remote administration, and (3) comprehensive content. First, the examination should not be burdensome, especially in fatigued patients with COVID-19 and their caregivers. Second, the examination must be conducted through remote communication, such as by telephone or a video telehealth session, that observes patient and caregiver distancing. Third, the examination should be capable of assessing mental functions related to specific brain structures in a short span of time.

Over the past several years, in consulting on patients admitted to medical and surgical inpatient units, we developed a 5-minute cognitive examination [5] adapted and extended from other instruments [6,7] that can characterize a panoply of brain disorders. These disorders include subtle forms of hepatic encephalopathy. Neuroanatomically, the examination tests temporal lobe memory functions, parietal lobe calculation ability, and each of the three frontosubcortical tracks that mediate (1) anterior cingulate engagement versus indifference, (2) anterior frontal lobe judgment, and (3) dorsolateral prefrontal executive functions [8,9]. The examination also assesses a series of standard mental status functions, such as basic orientation, concentration, fund of information, and ability to abstract meaning from concrete details. This brief examination can be expanded upon when responses indicate that further investigation is necessary.

Although most items on the examination can be found in other standardized formats, we have added two items that are particularly clinically useful. The first item is an ascending series of addition problems requiring mental calculation. Mathematics problems test parietal lobe functioning. The second item makes use of the Verbal Trails B Test in its entirety; this is a timed, standardized test of frontal executive functions [8] with a statistically derived cutoff point determining normal versus abnormal test responses.

Viewed as a quality assurance exercise, the purpose of this study was to afford early, proof-of-concept clinical data on the use and clinical implications of this 5-minute cognitive assessment with respect to patients with COVID-19. Here, we begin more rigorously to establish the validity of this assessment by providing clear-face value demonstrations of its external validity.

## Methods

**Figure 1:**
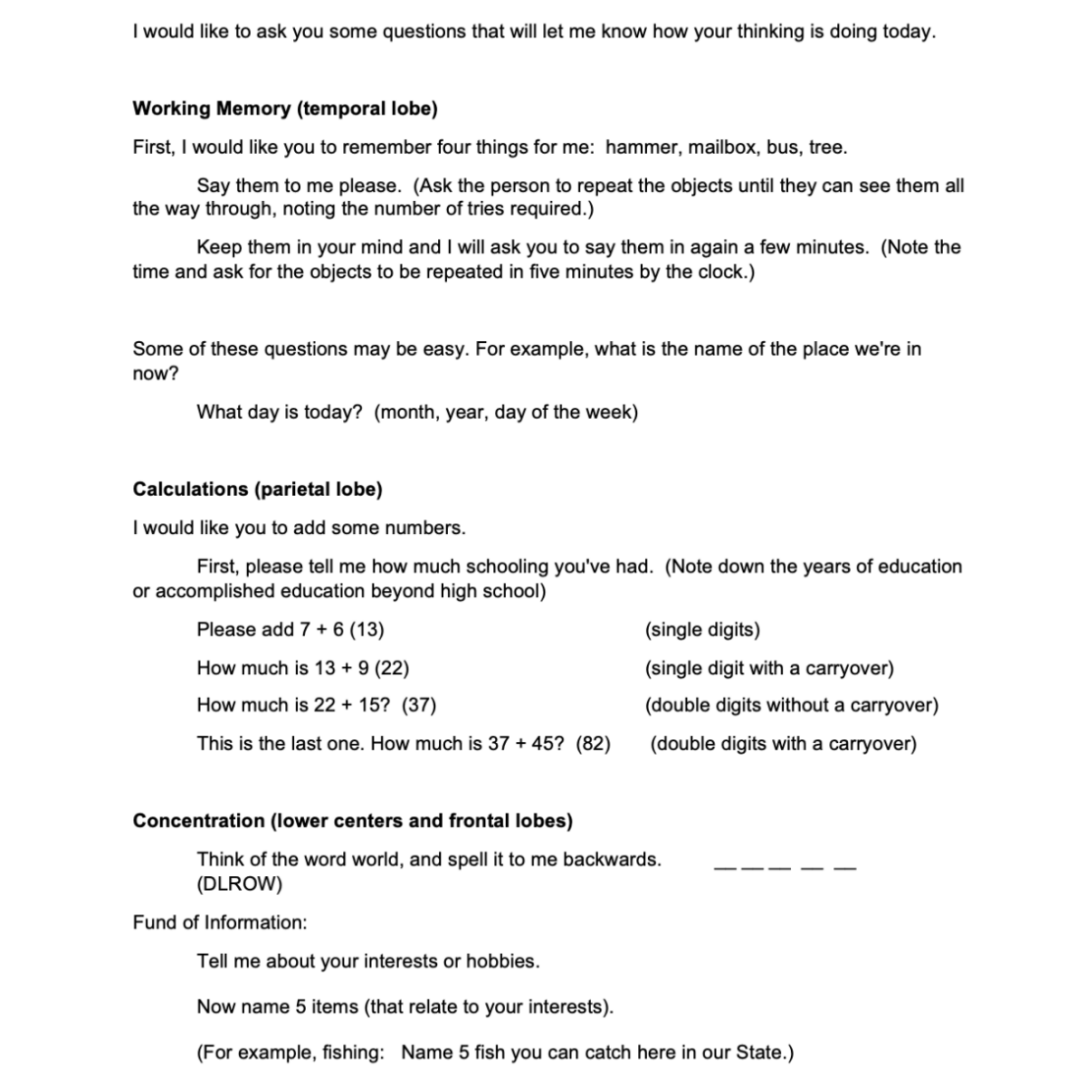
Part 1 of the brief cognitive examination for remote use (working memory, calculations, and concentration).

**Figure 2:**
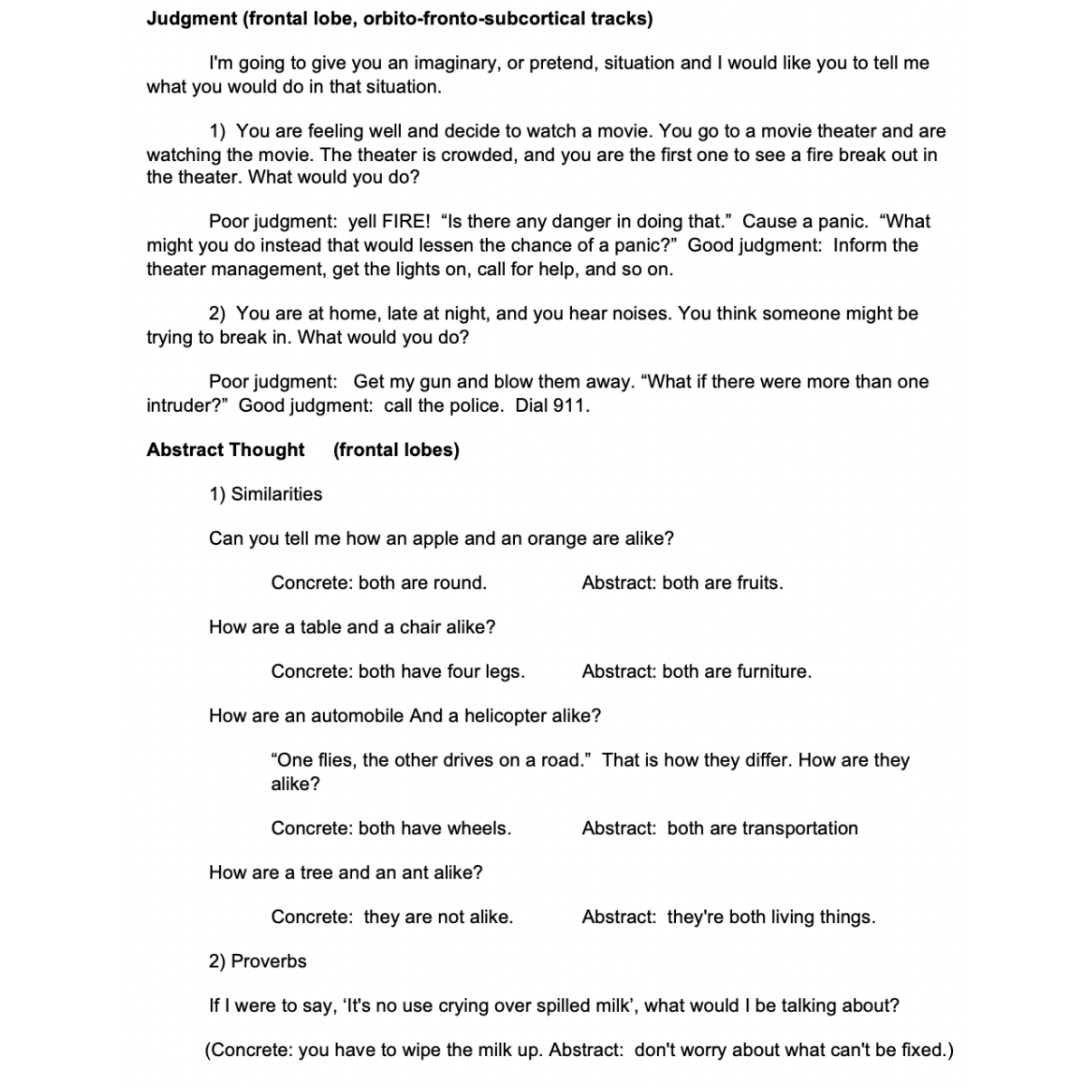
Part 2 of the brief cognitive examination for remote use (judgment and abstract thought).

**Figure 3:**
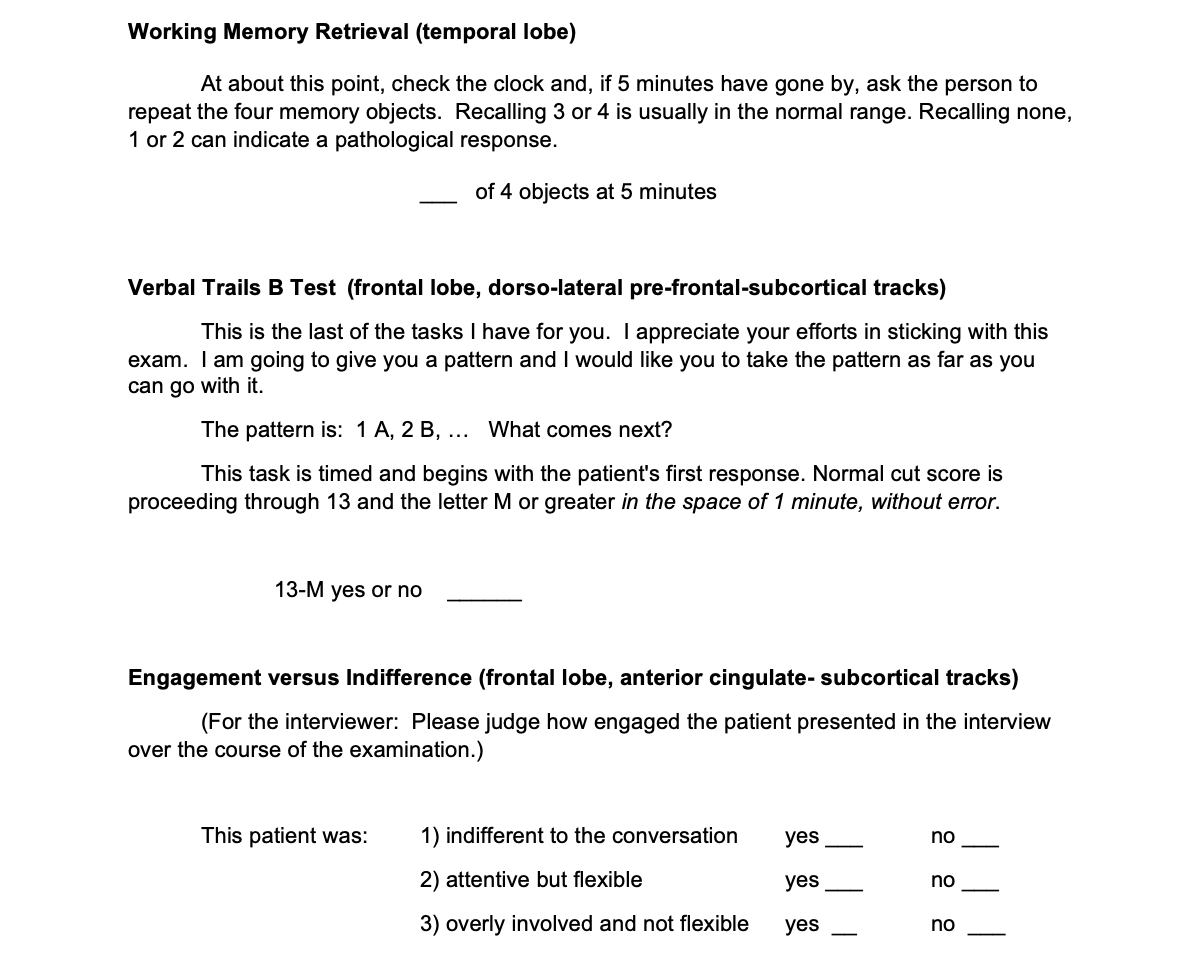
Part 3 of the brief cognitive examination for remote use (working memory retrieval, verbal trails B text, and engagement versus indifference).

We report here a series of 6 patients admitted for COVID-19 treatment to illustrate our experience with this telephone-based cognitive assessment. These case studies simply demonstrate the use of the assessment and do not quantitatively assess its validity or reliability. All patients were admitted for respiratory or fatigue symptoms to our Department of Veterans Affairs Medical Center, a large tertiary hospital that shares a campus with its university affiliate hospital. Examinations were conducted remotely during periods of hospital quarantine. For this review, all patients in this convenience sample were male and aged between 40 and 90 years. The same psychiatric consultant (TB) delivered each brief cognitive examination, as presented in Figures 1-3. Results were abstracted from our hospital records with Institutional Review Board approval. The cases have been disguised to preserve confidentiality. For brevity, we have included only details that are pertinent to assessing cognition. Our general hypothesis stated that we would find evidence of impaired cognition among patients with COVID-19.

## Results

### Case #1

This male patient, aged in his 70s, was admitted with shortness of breath, fever, chills, dry cough and malaise for 2 days. On Day 3, his nurses reported profound malaise and new onset of confusion. On Days 2-5, he received dexamethasone 6 mg daily. Psychiatry consultation on Day 5 revealed marked difficulty sustaining attention, a relative indifference to his medical condition, and an inability to understand simple directions. By Day 10, the patient was awake, alert, and attentive. His mood was one of relief with appropriate affect. His speech was fluent and logical. Other than fleeting shadows in his room, he experienced no hallucinations. He had lost the senses of both taste and smell.

Responses to the patient’s Day 10 telephone cognition assessment are listed below:

- Orientation: person, place, and date
- Memory: registered 4 objects, recalled 3 out of 4 after 5 minutes
- Calculations: complex addition intact (37 + 45 = 82)
- Concentration: “DLROW”
- Fund of information: the patient, a book collector, named 5 standard authors
- Judgment:
  fire-theater: “Contact help by phone or the theater personnel.”
  burglar noises at night: “Call 911.”
- Similarities/proverb:
  similarities: 3/3 abstract
  proverb: abstract
- Verbal Trails B [10]: lost track after 6-F

### Case #2

This male patient with schizophrenia, aged in his 60s, stable on clozapine for many years, presented with increasing confusion and profound malaise, and he had not taken his clozapine for approximately the past week. The admission diagnosis was COVID-19 pneumonia; the patient was awake but poorly responsive to conversation. On Day 2, he could not focus his attention for testing and was unable to use his phone. On Day 3, a 3-week clozapine up-taper began at 50 mg/day, gradually increasing to his regular dose of 450 mg/day, while monitoring neutrophil counts.

By Day 6, the patient was awake, alert, and able to converse by telephone. Responses to the assessment on Day 6 are listed below:

- Mood: calm and upbeat
- Affect: appropriate
- Speech: logical progressions
- Hallucinations/delusions: none endorsed on specific inquiry
- Orientation: person, place, and date
- Memory: registered 4 objects and recalled 3 at 5 minutes
- Calculation: unable to add double digits with carryover but could identify right/left and the fingers of both hands
- Concentration: “DROLW”
- Judgment: appropriate answers to fire-theater and burglar-noises, getting help in both cases
- Similarities/proverb:
  Similarities: 3/3 concrete
  proverb: concrete (“It’s about crying.”)
- Verbal Trails B: 10-J and then lost track

### Case #3

This male patient, aged in his 50s, was admitted with COVID-19 pneumonia; by Day 3, he required oxygen and was given dexamethasone. The nursing staff found him incontinent, with soiled clothes, to which he was indifferent, saying “I feel fine.” He needed assistance in showering and in understanding what the water knobs were for. During a telephone cognitive examination, he reported his mood as “good,” and he did not recall his confusion in the shower. He did not endorse hallucinations or paranoia. His thought process regarding similarities and a proverb was very concrete. Responses to the assessment are listed below:

- Orientation: to self and “hospital”
- Memory: could not register 4 objects on 3 tries
- Calculation: added single digit numbers only
- Verbal Trails B: could not understand the test directions

Responses to the Day 9 examination are listed below:

- Orientation: person, place, and date
- Memory: registered 4 items, recalled 3 after 5 minutes
- Calculation: added 2-digit numbers without carryover but not with carryover on 3 tries
- Judgment: fire-theater: “I'd leave the theater.” No further insight as to danger
- Similarities/proverb:
  Similarities: 3/3 concrete
  proverb: concrete (“You have to wipe the milk up.”)
- Verbal Trails B: unable to grasp the test directions on 3 tries

### Case #4

This male patient, aged in his 40s, with a history of polydrug abuse was referred from another hospital with a positive COVID-19 test during treatment for peripheral cellulitis and “minimal” COVID-19 symptoms. On Day 5, he developed psychosis with paranoia, disorganized speech, hyper-religiosity, mood lability, and insomnia. These symptoms required increasing haloperidol doses over 2 days to 10 mg/day. There was no hyperactivity, and the patient complained of profound fatigue.

Responses to the telephone evaluation on Day 5 are listed below:

- Orientation: person, place, and date
- Memory: registered 4 objects and recalled 3 at 5 minutes
- Calculation:
  unable to add 2-digit numbers
  stereognosis and finger naming were clear
- Concentration:
  “DLOW”
  did not see the error
- Fund: named 5 Super Bowl–winning teams
- Judgment: fire-theater: “Run out, call 911, run back in and get people out. Break down a wall if I had to.”
- Similarities/proverb:
  similarities: 3/3 abstract
  proverb: concrete (“Drink what's left.”)
- Verbal Trails B:
  grasped the instructions on the second try
  lost the sequence after 5-E

### Case #5

This male patient, aged in his 80s, was COVID-19–positive and admitted with “altered mental status,” malaise requiring oxygen, and supportive care with gradual improvement over 1 week. Responses to the Day 8 telephone assessment are listed below:

- Mood: “pretty good.”
- Hallucinations/delusions or suicidal concerns: none endorsed
- Speech: fluent and logical if somewhat slowed
- Orientation: person, place only
- Memory: registered 4 objects, recalled 2 at 5 minutes
- Calculation: added single digits with carryover only
- Concentration: “DOWOLW”
- Judgment: fire-theater: “Don’t yell FIRE!”; saw the panic danger but no alternative actions
- Verbal Trails B: lost track after 9-I

### Case #6

This male patient, aged in his 50s, was admitted with COVID-19 and malaise but no respiratory compromise. Responses to the Day 2 telephone assessment are listed below:

- Mood: demoralized state with slow, sparse speech
- Hallucinations:
  deprecatory voices
  “shadows, like ghosts” intermittently
- Orientation: person, place, and date
- Memory: registered 4 objects, recalled none at 5 minutes
- Verbal Trails B: unable to grasp the directions on 2 tries

On Day 7, the patient’s fatigue lifted. Responses to the Day 7 assessment are listed below:

- Mood: no demoralization
- Speech: fluent and logical
- Hallucinations: occasional nondeprecatory voices
- Orientation: person, place, and date
- Memory:
  registered 4 objects, recalled 1 of 4 objects at 5 minutes
  digit repetition: 5 numbers forward, not 6 on 2 tries
- Concentration: “DLROW”
- Calculations: unable to add double digits with or without carryover
- Fund of information: named 5 musicians he admires
- Judgment:
  fire-theater: “Yell FIRE!”; did not see the panic danger or appropriate actions
  burglar noises at night: “Call 911.”
- Similarities/proverb:
  similarities: 3/3 abstract
  proverb: abstract
- Verbal Trails B: understood the directions but lost track at 6-F

### Overall Results

Table 1 presents a collated listing of the cognitive presentations of the case patients. On admission, none of the patients were capable of meaningful engagements with the clinical staff; 5 were awake, if confused, and confusion with psychosis developed in the sixth patient (Case #4). This finding suggests involvement of the anterior cingulate cortex, which mediates engagement with one’s surroundings versus indifference to them [8,9]. This changed in the range of 1 week, as demonstrated by further assessment.

By that time, working memory had returned in 4 cases, indicating temporal lobe/hippocampal recovery. Judgment, which is associated with the anterior medial frontal lobe, returned to an extent in 3 cases, remaining poorly functional in 3. By contrast, 5 of the cases could not perform the ascending addition tasks, and 0 of the 6 performed normally on the Verbal Trails B Test. The latter finding is associated with impaired frontal executive function, such as planning and executing tasks. This impairment is associated with the dorsolateral prefrontal cortex.

Taken together, the case studies and Table 1 suggest a process of cognitive recovery over a short term of 5 to 10 days; however, questions on intermediate and long-term recovery remain unanswered. The data suggest the necessity of assessing higher cognitive functions that are associated with the neocortex in this brief format, with the goal of characterizing a series of cognitive functions differentially and in detail.

**Table 1:** Selected cognitive results of patients with COVID-19 (N=6).

Case #	Age (decade)	Examination day (hospitalization)	Temporal lobe: working memory (≥3 of 4 objects)	Parietal lobe: calculation (2 digits with carryover)	Frontal lobe engagement on Day 1	Frontal lobe: judgment (basic, complex)	Frontal lobe: Verbal Trails B (13-M without error)
1	70s	10	Yes	Correct	No	Complex	Poor
2	60s	6	Yes	No	No	Complex	Poor
3	50s	9	Yes	No	No	No	Poor
4	40s	5	Yes	No	No	No	Poor
5	80s	8	No	No	No	No	Poor
6	50s	7	No	No	No	Basic	Poor

## Discussion

As expected, this inquiry found specific evidence of cognitive impairments among this case series of patients with COVID-19. Unexpectedly, however, the differential characteristics of COVID-19 cognitive recovery appeared. We were surprised to observe that some cognitive components recovered more quickly than others. This observation raises questions regarding the timing and return of those items that appeared least likely to recover in the short term of 5 to 10 days, most notably parietal lobe calculation ability and frontal lobe executive functions. It also raises concerns over the recovery timing for temporal lobe working memory and frontal lobe judgment.

In the face of a neuropsychiatric pandemic [1], the principle clinical advantages of this examination lie in its ability to provide a rapid, brief assessment of several anatomically based cognitive functions in the least invasive manner possible to preserve both patient and caregiver safety. In our view, it offers a much wider array of easily administered tests compared to orientation alone or generic comments such as “altered mental state.” This examination offers a reasonable degree of brain function specificity and can be administered in approximately 5 minutes by telephone or videoconference.

This approach is not that of a screening examination that provides a series of tasks and delivers an overall score. Rather, it provides a first assessment of cognitive impairment that then directs further assessments in working through differential diagnoses toward a working or final diagnosis. For example, impaired working memory suggests the necessity to test registration functions further, such as through digit recall in Case #6, to clarify more generalized conditions such as memory impairment in delirium versus that in specific pathological conditions such as Wernicke-Korsakov Syndrome. Similarly, impaired calculation ability calls attention to the need to evaluate other parietal lobe functions, such as right/left orientation and finger agnosia, as in Cases #2 and #4.

This adapted format does not require a pencil and paper or in-person tasks; therefore, it is amenable to a telephone conversation. In contrast, the Frontal Assessment Battery (FAB) [11] must be performed in person, for example, when assessing the FAB’s judgment test of go/no-go responses. In contrast, the assessment reported here relies on assessing the cognitive process involved in judging what to do in a hypothetical situation.

Although the examination results are not diagnostic of specific causes [12], they do point to specific syndromes, such as delirium, that indicate further examination based on specific cognitive deficits. The syndromes themselves, including delirium, are frequently amenable to treatment, often with very low doses of antipsychotic agents such as haloperidol or olanzapine.

Although much more could be said about the increments of this 5-minute telephone cognitive assessment, its principal purpose is to characterize the nature and extent of impairment in patients with COVID-19 for both here-and-now assessment and follow-up recovery as the extent of illness lessens [4]. All the cases reported some form of cognitive impairment, whether profound, as in Cases #1 to #3, or more subtly, as in the other cases. Although all of the cases improved, 2 (#2 and #4) required antipsychotic medication to restore what was likely their preinfection baseline. Working memory, calculation ability, and especially Verbal Trails B performance—testing frontal executive abilities ascribed to dorsolateral prefrontal cortex functioning [8]—were most often impaired and slower to approach normal baseline capabilities. As our experience with COVID-19 develops, we suspect that longitudinal follow-up of brain functions will take on considerable import as we address recovery at pandemic population levels [12].

Beginning with a brief, multifaceted approach to recognizing cognitive impairment can open the way to more specific assessments and investigations. From this baseline, a longitudinal, prospective study can guide understanding of longer-term recovery. In the acute setting, as noted here, treatment trials targeting return of function, such as through low-dose neuroleptic agents aimed at cognitive dysfunction relief, can guide treatment efforts.

## Abbreviations

FAB: frontal assessment battery
NIMH: National Institute of Mental Health

## References

[1] Troyer EA, Kohn JN, Hong S (2020). Are we facing a crashing wave of neuropsychiatric sequelae of COVID-19? Neuropsychiatric symptoms and potential immunologic mechanisms. Brain Behav Immun.

[2] Lechien Jerome R, Chiesa-Estomba Carlos M, De Siati Daniele R, Horoi Mihaela, Le Bon Serge D, Rodriguez Alexandra, Dequanter Didier, Blecic Serge, El Afia Fahd, Distinguin Lea, Chekkoury-Idrissi Younes, Hans Stéphane, Delgado Irene Lopez, Calvo-Henriquez Christian, Lavigne Philippe, Falanga Chiara, Barillari Maria Rosaria, Cammaroto Giovanni, Khalife Mohamad, Leich Pierre, Souchay Christel, Rossi Camelia, Journe Fabrice, Hsieh Julien, Edjlali Myriam, Carlier Robert, Ris Laurence, Lovato Andrea, De Filippis Cosimo, Coppee Frederique, Fakhry Nicolas, Ayad Tareck, Saussez Sven (2020). Olfactory and gustatory dysfunctions as a clinical presentation of mild-to-moderate forms of the coronavirus disease (COVID-19): a multicenter European study. Eur Arch Otorhinolaryngol.

[3] Varatharaj A, Thomas Naomi, Ellul Mark A, Davies Nicholas W S, Pollak Thomas A, Tenorio Elizabeth L, Sultan Mustafa, Easton Ava, Breen Gerome, Zandi Michael, Coles Jonathan P, Manji Hadi, Al-Shahi Salman Rustam, Menon David K, Nicholson Timothy R, Benjamin Laura A, Carson Alan, Smith Craig, Turner Martin R, Solomon Tom, Kneen Rachel, Pett Sarah L, Galea Ian, Thomas Rhys H, Michael Benedict D, CoroNerve Study Group (2020). Neurological and neuropsychiatric complications of COVID-19 in 153 patients: a UK-wide surveillance study. Lancet Psychiatry.

[4] Oldham MA, Slooter AJC, Cunningham C, Rahman S, Davis D, Vardy ERLC, Garcez FB, Neufeld KJ, de Castro REV, Ely EW, MacLullich A (2020). Characterising neuropsychiatric disorders in patients with COVID-19. Lancet Psychiatry.

[5] Beresford T, Lucey Michael R (2018). Towards standardizing the alcoholism evaluation of potential liver transplant recipients. Alcohol Alcohol.

[6] Castanho TC, Amorim L, Zihl J, Palha JA, Sousa N, Santos NC (2014). Telephone-based screening tools for mild cognitive impairment and dementia in aging studies: a review of validated instruments. Front Aging Neurosci.

[7] Newkirk LA, Kim JM, Thompson JM, Tinklenberg JR, Yesavage JA, Taylor JL (2004). Validation of a 26-point telephone version of the Mini-Mental State Examination. J Geriatr Psychiatry Neurol.

[8] Arciniegas DB, Beresford TP (2001). Neuropsychiatry: An Introductory Approach.

[9] Arciniegas DB, Arciniegas DB, Anderson CA, Filley CM (2013). Chapter 23: Mental status examiniation. Behavioral Neurology & Neuropsychiatry.

[10] Tombaugh T (2004). Trail Making Test A and B: Normative data stratified by age and education. Arch Clin Neuropsychol.

[11] Dubois B, Slachevsky A, Litvan I, Pillon B (2000). The FAB: a Frontal Assessment Battery at bedside. Neurology.

[12] Paterson R, Brown Rachel L, Benjamin Laura, Nortley Ross, Wiethoff Sarah, Bharucha Tehmina, Jayaseelan Dipa L, Kumar Guru, Raftopoulos Rhian E, Zambreanu Laura, Vivekanandam Vinojini, Khoo Anthony, Geraldes Ruth, Chinthapalli Krishna, Boyd Elena, Tuzlali Hatice, Price Gary, Christofi Gerry, Morrow Jasper, McNamara Patricia, McLoughlin Benjamin, Lim Soon Tjin, Mehta Puja R, Levee Viva, Keddie Stephen, Yong Wisdom, Trip S Anand, Foulkes Alexander J M, Hotton Gary, Miller Thomas D, Everitt Alex D, Carswell Christopher, Davies Nicholas W S, Yoong Michael, Attwell David, Sreedharan Jemeen, Silber Eli, Schott Jonathan M, Chandratheva Arvind, Perry Richard J, Simister Robert, Checkley Anna, Longley Nicky, Farmer Simon F, Carletti Francesco, Houlihan Catherine, Thom Maria, Lunn Michael P, Spillane Jennifer, Howard Robin, Vincent Angela, Werring David J, Hoskote Chandrashekar, Jäger Hans Rolf, Manji Hadi, Zandi Michael S (2020). The emerging spectrum of COVID-19 neurology: clinical, radiological and laboratory findings. Brain.

